# Supplementary material for: The Local Tumor Microbiome Is Associated with Survival in Late-Stage Colorectal Cancer Patients
Source: Microbiol Spectr. 2023 Apr 12;11(3):e05066-22. doi: 10.1128/spectrum.05066-22 (PMC10269740; doi:10.1128/spectrum.05066-22)
Supplement: Supplemental file 1 — Supplemental material. Download spectrum.05066-22-s0001.pdf, PDF file, 1.6 MB [file spectrum.05066-22-s0001.pdf]

Supplemental tables

Table S1. Patient characteristics in the cohort
Table S2. Per-Subject Predictors of the Microbiome in CTF ordination space
Table S3. ASVs separating tumor and normal tissue based on differential ranking
Table S4. Effect of tissue type on beta diversity in an unpaired analysis
Table S5. Dissimilarity between normal and tumor tissue and survival
Table S6. Feature Ranks for tumor vs normal tissue, based on long survival
Table S7. Feature Ranks for interaction term (short survival in tumor tissue)
Table S8. Tumor rPCA PC 1 features-associated ASVS for the ALR calculation
Table S9. Tumor rPCA PC 2 associated features used in the ALR calculation
Table S10. STORMS check list

Supplemental Figures

Figure S1. A family level view of the microbiome
Figure S2. There is a strong individual effect on the microbiome
Figure S3. Metadata predictors of the microbiome in Compositional Tensor Factorization
Figure S4. Subject aware techniques detect a consistent difference between tissue types and replicate associated taxa
Figure S4. Survival separates individuals by tumor-associated rPCA quadrant

Deleted:
Deleted: Microbior
Deleted: tissue ba
Delet micro
Delet effect
Delet micro
Delet by tur

Table S1. Patient characteristics in the cohort

	Short-term survival (< 2 years) (n=50)	Long-term survival (≥5 years) (n=51)	Total (n=101)
Patient characteristics at time of surgery			
**Age, years**			
<60	6 (12.0)	9 (17.7)	15 (14.9)
60-69	11 (22.0)	19 (37.3)	30 (29.7)
70-74	16 (32.0)	10 (19.6)	26 (25.7)
≥75	17 (34.0)	13 (25.5)	30 (29.7)
**Sex**			
Female	24 (48.0)	21 (41.2)	45 (44.6)
Male	26 (52.0)	30 (58.8)	56 (55.5)
**ASA score**			
I (healthy)	14 (28.0)	15 (29.4)	29 (28.7)
II (mild)	21 (42.0)	28 (54.9)	49 (48.5)
III-IV (severe or worse)	15 (30.0)	8 (15.7)	23 (22.8)
**Pre-operative treatment**			
None	41 (82.0)	35 (68.6)	76 (75.3)
Radiotherapy	9 (18.0)	16 (31.4)	25 (24.7)
Tumor characteristics			
**Localization**			
Colon right	18 (36.0)	13 (25.5)	31 (30.7)
Colon left	11 (22.0)	17 (33.3)	28 (27.7)
Rectum	21 (42.0)	21 (41.2)	42 (41.6)
**Mucinous cancer**			
no	45 (90.0)	45 (88.2)	90 (89.1)
yes	5 (10.0)	6 (11.8)	11 (10.9)
**TNM stage**			
III	23 (46.0)	46 (90.2)	69 (68.3)
IV	27 (54.0)	5 (9.8)	32 (31.7)
**Grade of differentiation**			
low	22 (44.0)	7 (13.7)	29 (28.7)
medium	24 (48.0)	38 (74.5)	62 (61.4)
high	4 (8.0)	6 (11.8)	10 (9.9)
Surgical characteristics			
**Period of surgery**			
1997-2005	19 (38.0)	13 (25.5)	32 (31.7)
2006-2010	18 (36.0)	23 (45.1)	41 (40.6)
2011-2017	13 (26.0)	15 (29.4)	28 (27.7)
**Radical operation**			
no	14 (28.0)	1 (2.0)	15 (14.9)
yes	36 (72.0)	50 (98.0)	86 (85.2)
**Microscopic radical operation**			
no	13 (26.9)	1 (2.0)	14 (13.9)
yes	37 (74.0)	50 (98.0)	87 (86.1)

Table S2. Per-Subject Predictors of the Microbiome in CTF ordination space

Predictor	Variation explained (%)	Crude p-value (999 permutations)
Age, years	0.020	0.776
Sex	0.011	0.364
ASA score	0.034	0.140
Localization	0.011	0.778
TNM Stage	0.004	0.764
Grade of differentiation	0.007	0.931
Period of Surgery	0.151	0.001
Survival	0.020	0.113

Table S3. ASVs separating tumor and normal tissue based on differential ranking

		ASV ID	Prevalence		Taxonomy
			ASV	Cumulative	
Normal tissue associated	1	Clos-b4ed8ec	27.7%	27.7%	p. Firmicutes; c. Clostridia; o. Clostridiales; f. Clostridiaceae 1; g. Clostridium sensu stricto 1
	2	Lach-e23e6e7	21.8%	39.6%	p. Firmicutes; c. Clostridia; o. Clostridiales; f. Lachnospiraceae; g. uncl f. Lachnospiraceae
	3	Bact-7ce20dc	19.8%	50.0%	p. Bacteroidetes; c. Bacteroidia; o. Bacteroidales; f. Bacteroidaceae; g. Bacteroides
	4	Marv-c55b486	36.1%	68.8%	p. Firmicutes; c. Clostridia; o. Clostridiales; f. Lachnospiraceae; g. Marvinbryantia
	5	Bact-47d7490	18.8%	73.3%	p. Bacteroidetes; c. Bacteroidia; o. Bacteroidales; f. Bacteroidaceae; g. Bacteroides
	6	Pept-1d2b00f	19.8%	74.8%	p. Firmicutes; c. Clostridia; o. Clostridiales; f. Peptostreptococcaceae; g. Peptostreptococcus
	7	Para-3d48874	21.3%	79.7%	p. Bacteroidetes; c. Bacteroidia; o. Bacteroidales; f. Prevotellaceae; g. Paraprevotella
	8	Rumi-0f06bc7	24.8%	82.2%	p. Firmicutes; c. Clostridia; o. Clostridiales; f. Ruminococcaceae; g. Ruminococcaceae UCG-002
	9	Lach-11902be	38.1%	89.6%	p. Firmicutes; c. Clostridia; o. Clostridiales; f. Lachnospiraceae; g. uncl f. Lachnospiraceae
	10	Rumi-3efbc4a	29.2%	90.6%	p. Firmicutes; c. Clostridia; o. Clostridiales; f. Ruminococcaceae; g. uncl f. Ruminococcaceae
	11	Alis-520887a	30.7%	92.1%	p. Bacteroidetes; c. Bacteroidia; o. Bacteroidales; f. Rikenellaceae; g. Alistipes
	12	Bact-87e7824	15.3%	93.1%	p. Bacteroidetes; c. Bacteroidia; o. Bacteroidales; f. Bacteroidaceae; g. Bacteroides
	13	Buty-da8b26f	43.6%	96.0%	p. Firmicutes; c. Clostridia; o. Clostridiales; f. Ruminococcaceae; g. Butyricicoccus
	14	Anae-4f9106b	30.7%	97.0%	p. Firmicutes; c. Clostridia; o. Clostridiales; f. Ruminococcaceae; g. Anaerotruncus
	15	Rumi-494e655	33.7%	97.0%	p. Firmicutes; c. Clostridia; o. Clostridiales; f. Ruminococcaceae; g. Ruminococcaceae UCG-002
	16	Anae-2ee585c	26.2%	97.5%	p. Firmicutes; c. Clostridia; o. Clostridiales; f. Lachnospiraceae; g. Anaerostipes
	17	Blau-400ef32	64.4%	98.0%	p. Firmicutes; c. Clostridia; o. Clostridiales; f. Lachnospiraceae; g. Blautia
	18	Bifi-38b7580	41.6%	98.0%	p. Actinobacteria; c. Actinobacteria; o. Bifidobacteriales; f. Bifidobacteriaceae; g. Bifidobacterium
	19	Rumi-a39d801	19.3%	99.0%	p. Firmicutes; c. Clostridia; o. Clostridiales; f. Ruminococcaceae; g. Ruminococcus 2
	20	Rumi-75a7dd0	24.8%	99.0%	p. Firmicutes; c. Clostridia; o. Clostridiales; f. Ruminococcaceae; g. Ruminococcus 2
	21	Rumi-f7d40e3	20.3%	99.5%	p. Firmicutes; c. Clostridia; o. Clostridiales; f. Ruminococcaceae; g. Ruminococcus 2
	22	Blau-8f6e2a9	95.0%	100.0%	p. Firmicutes; c. Clostridia; o. Clostridiales; f. Lachnospiraceae; g. Blautia
Tumor Tissue Associated	1	Fuso-e47b7c5	25.2%	25.2%	p. Fusobacteria; c. Fusobacteriia; o. Fusobacteriales; f. Fusobacteriaceae; g. Fusobacterium
	2	Camp-5b14d87	27.2%	46.5%	p. Proteobacteria; c. Epsilonproteobacteria; o. Campylobacterales; f. Campylobacteraceae; g. Campylobacter
	3	Fuso-d2f4ba7	12.4%	50.0%	p. Fusobacteria; c. Fusobacteriia; o. Fusobacteriales; f. Fusobacteriaceae; g. Fusobacterium
	4	Hung-78c2b8e	33.7%	61.9%	p. Firmicutes; c. Clostridia; o. Clostridiales; f. Lachnospiraceae; g. Hungatella
	5	Fuso-7fa9543	21.3%	66.8%	p. Fusobacteria; c. Fusobacteriia; o. Fusobacteriales; f. Fusobacteriaceae; g. Fusobacterium
	6	Gran-a5b69f1	42.6%	79.7%	p. Firmicutes; c. Bacilli; o. Lactobacillales; f. Carnobacteriaceae; g. Granulicatella
	7	Bact-26547c9	34.2%	87.6%	p. Bacteroidetes; c. Bacteroidia; o. Bacteroidales; f. Bacteroidaceae; g. Bacteroides
	8	Stre-d3829c5	19.3%	88.1%	p. Firmicutes; c. Bacilli; o. Lactobacillales; f. Streptococcaceae; g. Streptococcus
	9	Mass-bfb5b51	38.1%	91.6%	p. Proteobacteria; c. Betaproteobacteria; o. Burkholderiales; f. Oxalobacteraceae; g. Massilia
	10	Para-8d83490	11.9%	92.6%	p. Bacteroidetes; c. Bacteroidia; o. Bacteroidales; f. Porphyromonadaceae; g. Parabacteroides
	11	Porp-69023d0	18.8%	92.6%	p. Bacteroidetes; c. Bacteroidia; o. Bacteroidales; f. Porphyromonadaceae; g. Porphyromonas
	12	Lach-7cd4efa	54.0%	95.5%	p. Firmicutes; c. Clostridia; o. Clostridiales; f. Lachnospiraceae; g. Lachnoclostridium
	13	Hung-82c448c	29.7%	96.0%	p. Firmicutes; c. Clostridia; o. Clostridiales; f. Lachnospiraceae; g. Hungatella
	14	Para-98a6938	17.3%	97.5%	p. Proteobacteria; c. Betaproteobacteria; o. Burkholderiales; f. Alcaligenaceae; g. Parasutterella
	15	Stre-101637a	36.6%	98.5%	p. Firmicutes; c. Bacilli; o. Lactobacillales; f. Streptococcaceae; g. Streptococcus
	16	Sutt-1d5d65b	19.3%	99.5%	p. Proteobacteria; c. Betaproteobacteria; o. Burkholderiales; f. Alcaligenaceae; g. Sutterella
	17	Rumi-eee1408	20.3%	100.0%	p. Firmicutes; c. Clostridia; o. Clostridiales; f. Ruminococcaceae; g. Ruminococcaceae UCG-014

Table S4. Effect of tissue type on beta diversity in an unpaired analysis

	Our Data		Flemer *et al* (replication)	
**Metric**	Variance Explained (%)	Crude p-value (999 permutations)	Variance Explained (%)	Crude p-value (999 permutations)
Bray Curtis dissimilarity	0.34	0.993	0.57	0.999
Jaccard distance	0.24	1.000	0.79	1.000
Unweighted UniFrac distance	0.39	0.887	--	--
Weighted UniFrac distance	0.67	0.164	--	--

Table S5. Difference between normal and tumor tissue and survival

Metric	Crude	Fully adjusted[1]
Binary Jaccard (0.1 unit)	1.53 (1.00, 2.34)	1.61 (0.83, 3.15)
Aitchison (10 units)	1.91 (1.12, 3.27)	1.42 (0.59, 3.44)
CTF (1 unit)	1.20 (0.54, 2.67)	1.94 (0.54, 6.98)
Bray Curtis (0.1 unit)	1.63 (1.18, 2.26)	1.70 (1.06, 2.73)
unweighted UniFrac (0.1 unit)	1.91 (1.06, 3.43)	2.01 (0.80, 5.02)
weighted UniFrac (0.1 unit)	1.36 (1.11, 1.66)	1.48 (1.08, 2.03)

[1]Adjusted for age, sex, ASA score, tumor localization, TNM stage, differentiation grade, surgery period, and radical surgery

Table S6. Feature Ranks for tumor vs normal tissue, based on long survival

		Feature-id	Prevalence		Taxonomy
			ASV	Cumulative	
Normal tissue associated	1	Bact-47d7490	18.8%	18.8%	p. Bacteroidetes; c. Bacteroidia; o. Bacteroidales; f. Bacteroidaceae; g. Bacteroides
	2	Pept-1d2b00f	19.8%	34.2%	p. Firmicutes; c. Clostridia; o. Clostridiales; f. Peptostreptococcaceae; g. Peptostreptococcus
	3	Bact-208134d	17.8%	45.5%	p. Bacteroidetes; c. Bacteroidia; o. Bacteroidales; f. Bacteroidaceae; g. Bacteroides
	4	Para-3d48874	21.3%	52.5%	p. Bacteroidetes; c. Bacteroidia; o. Bacteroidales; f. Prevotellaceae; g. Paraprevotella
	5	Bact-5859c64	25.2%	64.4%	p. Bacteroidetes; c. Bacteroidia; o. Bacteroidales; f. Bacteroidaceae; g. Bacteroides
	6	Barn-f674778	19.3%	70.8%	p. Bacteroidetes; c. Bacteroidia; o. Bacteroidales; f. Porphyromonadaceae; g. Barnesiella
	7	Marv-c55b486	36.1%	80.2%	p. Firmicutes; c. Clostridia; o. Clostridiales; f. Lachnospiraceae; g. Marvinbryantia
	8	Clos-b4ed8ec	27.7%	84.2%	p. Firmicutes; c. Clostridia; o. Clostridiales; f. Clostridiaceae 1; g. Clostridium sensu stricto 1
	9	Para-218f40f	35.6%	89.6%	p. Bacteroidetes; c. Bacteroidia; o. Bacteroidales; f. Porphyromonadaceae; g. Parabacteroides
	10	Bifi-38b7580	41.6%	92.1%	p. Actinobacteria; c. Actinobacteria; o. Bifidobacteriales; f. Bifidobacteriaceae; g. Bifidobacterium
	11	Desu-fa6956a	23.3%	93.1%	p. Proteobacteria; c. Deltaproteobacteria; o. Desulfovibrionales; f. Desulfovibrionaceae; g. Desulfovibrio
	12	Meth-26639d9	24.8%	93.1%	p. Euryarchaeota; c. Methanobacteria; o. Methanobacteriales; f. Methanobacteriaceae; g. Methanobrevibacter
	13	Lach-e23e6e7	21.8%	93.1%	p. Firmicutes; c. Clostridia; o. Clostridiales; f. Lachnospiraceae; g. unsp. f. Lachnospiraceae
	14	Bact-b74ab69	19.3%	94.1%	p. Bacteroidetes; c. Bacteroidia; o. Bacteroidales; f. Bacteroidaceae; g. Bacteroides
	15	Stre-a4cd615	16.8%	95.5%	p. Firmicutes; c. Bacilli; o. Lactobacillales; f. Streptococcaceae; g. Streptococcus
	16	Sutt-381ef54	19.8%	96.0%	p. Proteobacteria; c. Betaproteobacteria; o. Burkholderiales; f. Alcaligenaceae; g. Sutterella
	17	Bact-7ce20dc	19.8%	96.0%	p. Bacteroidetes; c. Bacteroidia; o. Bacteroidales; f. Bacteroidaceae; g. Bacteroides
	18	Buty-da8b26f	43.6%	97.0%	p. Firmicutes; c. Clostridia; o. Clostridiales; f. Ruminococcaceae; g. Butyricicoccus
	19	Rumi-4a1547a	46.5%	97.0%	p. Firmicutes; c. Clostridia; o. Clostridiales; f. Ruminococcaceae; g. Ruminiclostridium 6
	20	Rumi-8dc9986	21.8%	98.0%	p. Firmicutes; c. Clostridia; o. Clostridiales; f. Ruminococcaceae; g. Ruminococcaceae UCG-002
	21	Bifi-f633cf9	24.8%	98.0%	p. Actinobacteria; c. Actinobacteria; o. Bifidobacteriales; f. Bifidobacteriaceae; g. Bifidobacterium
	22	Turi-c96cf26	25.2%	98.0%	p. Firmicutes; c. Erysipelotrichia; o. Erysipelotrichales; f. Erysipelotrichaceae; g. Turicibacter
	23	Mass-cabd729	24.8%	99.0%	p. Proteobacteria; c. Betaproteobacteria; o. Burkholderiales; f. Oxalobacteraceae; g. Massilia
	24	Rumi-75a7dd0	24.8%	99.0%	p. Firmicutes; c. Clostridia; o. Clostridiales; f. Ruminococcaceae; g. Ruminococcus 2
	25	Hold-5637d39	14.4%	99.0%	p. Firmicutes; c. Erysipelotrichia; o. Erysipelotrichales; f. Erysipelotrichaceae; g. Holdemanella
	26	Haem-18eb929	41.1%	99.5%	p. Proteobacteria; c. Gammaproteobacteria; o. Pasteurellales; f. Pasteurellaceae; g. Haemophilus
	27	Chri-b03de84	43.1%	100.0%	p. Firmicutes; c. Clostridia; o. Clostridiales; f. Christensenellaceae; g. Christensenellaceae R-7 group

		Feature-id	Prevalence		Taxonomy
			ASV	Cumulative	
Tumor tissue	1	Hung-78c2b8e	33.7%	33.7%	p. Firmicutes; c. Clostridia; o. Clostridiales; f. Lachnospiraceae; g. Hungatella
	2	Fuso-e47b7c5	25.2%	47.0%	p. Fusobacteria; c. Fusobacteriia; o. Fusobacteriales; f. Fusobacteriaceae; g. Fusobacterium
	3	Lach-7cd4efa	54.0%	66.8%	p. Firmicutes; c. Clostridia; o. Clostridiales; f. Lachnospiraceae; g. Lachnoclostridium
	4	Camp-5b14d87	27.2%	77.7%	p. Proteobacteria; c. Epsilonproteobacteria; o. Campylobacterales; f. Campylobacteraceae; g. Campylobacter
	5	Rose-3a89328	37.1%	86.1%	p. Firmicutes; c. Clostridia; o. Clostridiales; f. Lachnospiraceae; g. Roseburia
	6	Sutt-1d5d65b	19.3%	88.6%	p. Proteobacteria; c. Betaproteobacteria; o. Burkholderiales; f. Alcaligenaceae; g. Sutterella
	7	Stre-d3829c5	19.3%	91.1%	p. Firmicutes; c. Bacilli; o. Lactobacillales; f. Streptococcaceae; g. Streptococcus
	8	Buty-62c3369	25.2%	92.1%	p. Firmicutes; c. Clostridia; o. Clostridiales; f. Ruminococcaceae; g. Butyricicoccus
	9	Hung-82c448c	29.7%	93.6%	p. Firmicutes; c. Clostridia; o. Clostridiales; f. Lachnospiraceae; g. Hungatella
	10	Fuso-d2f4ba7	12.4%	93.6%	p. Fusobacteria; c. Fusobacteriia; o. Fusobacteriales; f. Fusobacteriaceae; g. Fusobacterium
	11	Mass-bfb5b51	38.1%	95.5%	p. Proteobacteria; c. Betaproteobacteria; o. Burkholderiales; f. Oxalobacteraceae; g. Massilia
	12	Bact-866ea1c	13.9%	95.5%	p. Bacteroidetes; c. Bacteroidia; o. Bacteroidales; f. Bacteroidaceae; g. Bacteroides
	13	Porp-0799969	21.3%	95.5%	p. Bacteroidetes; c. Bacteroidia; o. Bacteroidales; f. Porphyromonadaceae; g. Porphyromonas
	14	Dial-08dda4f	13.4%	96.0%	p. Firmicutes; c. Negativicutes; o. Selenomonadales; f. Veillonellaceae; g. Dialister
	15	Barn-53f976e	14.9%	97.5%	p. Bacteroidetes; c. Bacteroidia; o. Bacteroidales; f. Porphyromonadaceae; g. Barnesiella
	16	Bact-0246885	20.8%	97.5%	p. Bacteroidetes; c. Bacteroidia; o. Bacteroidales; f. Bacteroidaceae; g. Bacteroides
	17	Phas-fb7a1c0	20.8%	97.5%	p. Firmicutes; c. Negativicutes; o. Selenomonadales; f. Acidaminococcaceae; g. Phascolarctobacterium
	18	Rumi-6a57339	31.2%	98.0%	p. Firmicutes; c. Clostridia; o. Clostridiales; f. Ruminococcaceae; g. unsp. f. Ruminococcaceae
	19	Esch-ffc36e2	90.6%	100.0%	p. Proteobacteria; c. Gammaproteobacteria; o. Enterobacteriales; f. Enterobacteriaceae; g. Escherichia/Shigella

Table S7. Feature Ranks for interaction term (short survival in tumor tissue)

		Feature-id	Prevalence ASV	Prevalence Cumulative	Taxonomy
Normal Tissue	1	Chri-fb2e43a	20.3%	20.3%	p. Firmicutes; c. Clostridia; o. Clostridiales; f. Christensenellaceae; g. Christensenellaceae R-7 group
	2	Anae-2ee585c	26.2%	40.6%	p. Firmicutes; c. Clostridia; o. Clostridiales; f. Lachnospiraceae; g. Anaerostipes
	3	Alis-0ce6000	14.9%	50.5%	p. Bacteroidetes; c. Bacteroidia; o. Bacteroidales; f. Rikenellaceae; g. Alistipes
	4	Barn-53f976e	14.9%	55.9%	p. Bacteroidetes; c. Bacteroidia; o. Bacteroidales; f. Porphyromonadaceae; g. Barnesiella
	5	Chri-f7035cb	20.8%	63.9%	p. Firmicutes; c. Clostridia; o. Clostridiales; f. Christensenellaceae; g. Christensenellaceae R-7 group
	6	Romb-df3d611	55.0%	78.7%	p. Firmicutes; c. Clostridia; o. Clostridiales; f. Peptostreptococcaceae; g. Romboutsia
	7	Rumi-6c7b500	19.3%	80.7%	p. Firmicutes; c. Clostridia; o. Clostridiales; f. Ruminococcaceae; g. Ruminococcaceae NK4A214 group
	8	Rhod-1667bf7	24.3%	83.2%	p. Proteobacteria; c. Alphaproteobacteria; o. Rhodospirillales; f. Rhodospirillaceae; g. unsp. f. Rhodospirillaceae
	9	Lach-7079408	50.5%	88.1%	p. Firmicutes; c. Clostridia; o. Clostridiales; f. Lachnospiraceae; g. Lachnospiraceae NK4A136 group
	10	Rumi-6a57339	31.2%	91.6%	p. Firmicutes; c. Clostridia; o. Clostridiales; f. Ruminococcaceae; g. unsp. f. Ruminococcaceae
	11	Alis-520887a	30.7%	93.6%	p. Bacteroidetes; c. Bacteroidia; o. Bacteroidales; f. Rikenellaceae; g. Alistipes
	12	Sene-781430c	24.8%	93.6%	p. Actinobacteria; c. Coriobacteriia; o. Coriobacteriales; f. Coriobacteriaceae; g. Senegalimassilia
	13	Rumi-3efbc4a	29.2%	93.6%	p. Firmicutes; c. Clostridia; o. Clostridiales; f. Ruminococcaceae; g. unsp. f. Ruminococcaceae
	14	Buty-62c3369	25.2%	95.0%	p. Firmicutes; c. Clostridia; o. Clostridiales; f. Ruminococcaceae; g. Butyricicoccus
	15	Lach-7cd4efa	54.0%	97.0%	p. Firmicutes; c. Clostridia; o. Clostridiales; f. Lachnospiraceae; g. Lachnoclostridium
	16	Rose-3a89328	37.1%	99.0%	p. Firmicutes; c. Clostridia; o. Clostridiales; f. Lachnospiraceae; g. Roseburia
	17	Rose-d3864fc	34.2%	99.0%	p. Firmicutes; c. Clostridia; o. Clostridiales; f. Lachnospiraceae; g. Roseburia
	18	Lach-3a2e845	33.7%	99.5%	p. Firmicutes; c. Clostridia; o. Clostridiales; f. Lachnospiraceae; g. unsp. f. Lachnospiraceae
	19	Hung-78c2b8e	33.7%	100.0%	p. Firmicutes; c. Clostridia; o. Clostridiales; f. Lachnospiraceae; g. Hungatella

		Feature-id	Prevalence		Taxonomy
			ASV	Cumulative	
Tumor Tissue	1	Pept-1d2b00f	19.8%	19.8%	p. Firmicutes; c. Clostridia; o. Clostridiales; f. Peptostreptococcaceae; g. Peptostreptococcus
	2	Bact-47d7490	18.8%	34.2%	p. Bacteroidetes; c. Bacteroidia; o. Bacteroidales; f. Bacteroidaceae; g. Bacteroides
	3	Bifi-c299192	14.4%	45.0%	p. Actinobacteria; c. Actinobacteria; o. Bifidobacteriales; f. Bifidobacteriaceae; g. Bifidobacterium
	4	Bact-208134d	17.8%	55.9%	p. Bacteroidetes; c. Bacteroidia; o. Bacteroidales; f. Bacteroidaceae; g. Bacteroides
	5	Sutt-381ef54	19.8%	67.3%	p. Proteobacteria; c. Betaproteobacteria; o. Burkholderiales; f. Alcaligenaceae; g. Sutterella
	6	Para-3d48874	21.3%	71.3%	p. Bacteroidetes; c. Bacteroidia; o. Bacteroidales; f. Prevotellaceae; g. Paraprevotella
	7	Bact-5859c64	25.2%	77.2%	p. Bacteroidetes; c. Bacteroidia; o. Bacteroidales; f. Bacteroidaceae; g. Bacteroides
	8	Veil-24522f7	27.7%	81.7%	p. Firmicutes; c. Negativicutes; o. Selenomonadales; f. Veillonellaceae; g. Veillonella
	9	Barn-f674778	19.3%	85.1%	p. Bacteroidetes; c. Bacteroidia; o. Bacteroidales; f. Porphyromonadaceae; g. Barnesiella
	10	Bact-26547c9	34.2%	92.1%	p. Bacteroidetes; c. Bacteroidia; o. Bacteroidales; f. Bacteroidaceae; g. Bacteroides
	11	Chri-b03de84	43.1%	95.0%	p. Firmicutes; c. Clostridia; o. Clostridiales; f. Christensenellaceae; g. Christensenellaceae R-7 group
	12	Bact-98fcc7e	53.5%	96.0%	p. Bacteroidetes; c. Bacteroidia; o. Bacteroidales; f. Bacteroidaceae; g. Bacteroides
	13	Fuso-7fa9543	21.3%	96.0%	p. Fusobacteria; c. Fusobacteriia; o. Fusobacteriales; f. Fusobacteriaceae; g. Fusobacterium
	14	Sutt-222e21f	25.2%	97.0%	p. Proteobacteria; c. Betaproteobacteria; o. Burkholderiales; f. Alcaligenaceae; g. Sutterella
	15	Para-218f40f	35.6%	97.5%	p. Bacteroidetes; c. Bacteroidia; o. Bacteroidales; f. Porphyromonadaceae; g. Parabacteroides
	16	Bifi-f633cf9	24.8%	97.5%	p. Actinobacteria; c. Actinobacteria; o. Bifidobacteriales; f. Bifidobacteriaceae; g. Bifidobacterium
	17	Clos-875f9ef	27.2%	99.0%	p. Firmicutes; c. Clostridia; o. Clostridiales; f. Clostridiaceae 1; g. Clostridium sensu stricto 1
	18	Rose-d91b5c7	34.2%	99.5%	p. Firmicutes; c. Clostridia; o. Clostridiales; f. Lachnospiraceae; g. Roseburia
	19	Fret-2b8622a	22.3%	99.5%	p. Synergistetes; c. Synergistia; o. Synergistales; f. Synergistaceae; g. Fretibacterium
	20	Fuso-62b2f8b	19.3%	99.5%	p. Fusobacteria; c. Fusobacteriia; o. Fusobacteriales; f. Fusobacteriaceae; g. Fusobacterium
	21	Sutt-dfc8256	33.7%	99.5%	p. Proteobacteria; c. Betaproteobacteria; o. Burkholderiales; f. Alcaligenaceae; g. Sutterella
	22	Marv-c55b486	36.1%	99.5%	p. Firmicutes; c. Clostridia; o. Clostridiales; f. Lachnospiraceae; g. Marvinbryantia
	23	Rumi-4a1547a	46.5%	99.5%	p. Firmicutes; c. Clostridia; o. Clostridiales; f. Ruminococcaceae; g. Ruminiclostridium 6
	24	Bact-b74ab69	19.3%	99.5%	p. Bacteroidetes; c. Bacteroidia; o. Bacteroidales; f. Bacteroidaceae; g. Bacteroides
	25	Gast-90e9bc6	24.8%	99.5%	p. Cyanobacteria; c. Melainabacteria; o. Gastranaerophilales; f. unsp. o. Gastranaerophilales; g. unsp. o. Gastranaerophilales
	26	Meth-26639d9	24.8%	99.5%	p. Euryarchaeota; c. Methanobacteria; o. Methanobacteriales; f. Methanobacteriaceae; g. Methanobrevibacter
	27	Dial-35fd415	60.4%	99.5%	p. Firmicutes; c. Negativicutes; o. Selenomonadales; f. Veillonellaceae; g. Dialister

28	Lach-3765e25	30.2%	99.5%	p. Firmicutes; c. Clostridia; o. Clostridiales; f. Lachnospiraceae; g. Lachnospiraceae NK4A136 group
29	Desu-fa6956a	23.3%	99.5%	p. Proteobacteria; c. Deltaproteobacteria; o. Desulfovibrionales; f. Desulfovibrionaceae; g. Desulfovibrio
30	Rumi-8e121f6	38.1%	100.0%	p. Firmicutes; c. Clostridia; o. Clostridiales; f. Ruminococcaceae; g. unsp. f. Ruminococcaceae

Table S8. Tumor rPCA PC 1 features-associated ASVS for the ALR calculation

	Feature-id	Taxonomy
Long-term Survival	Bact-70d55ba	p. Bacteroidetes; c. Bacteroidia; o. Bacteroidales; f. Bacteroidaceae; g. Bacteroides
	Bact-b6635d6	p. Bacteroidetes; c. Bacteroidia; o. Bacteroidales; f. Bacteroidaceae; g. Bacteroides
	Faec-4516aa6	p. Firmicutes; c. Clostridia; o. Clostridiales; f. Ruminococcaceae; g. Faecalibacterium
	Faec-c728ad6	p. Firmicutes; c. Clostridia; o. Clostridiales; f. Ruminococcaceae; g. Faecalibacterium
	Faec-22f4ee9	p. Firmicutes; c. Clostridia; o. Clostridiales; f. Ruminococcaceae; g. Faecalibacterium
	Subd-c6fdf63	p. Firmicutes; c. Clostridia; o. Clostridiales; f. Ruminococcaceae; g. Subdoligranulum
	Rose-b264ac8	p. Firmicutes; c. Clostridia; o. Clostridiales; f. Lachnospiraceae; g. Roseburia
	Subd-b6a1cfc	p. Firmicutes; c. Clostridia; o. Clostridiales; f. Ruminococcaceae; g. Subdoligranulum
	Fusi-9df2517	p. Firmicutes; c. Clostridia; o. Clostridiales; f. Lachnospiraceae; g. Fusicatenibacter
	Bifi-3f3a0ea	p. Actinobacteria; c. Actinobacteria; o. Bifidobacteriales; f. Bifidobacteriaceae; g. Bifidobacterium
Short-term Survival	Pept-f148c98	p. Firmicutes; c. Clostridia; o. Clostridiales; f. Peptostreptococcaceae; g. Peptostreptococcus
	Bact-47f3d64	p. Bacteroidetes; c. Bacteroidia; o. Bacteroidales; f. Bacteroidaceae; g. Bacteroides
	Dial-63719d6	p. Firmicutes; c. Negativicutes; o. Selenomonadales; f. Veillonellaceae; g. Dialister
	Parv-8f3c8cb	p. Firmicutes; c. Clostridia; o. Clostridiales; f. Family XI; g. Parvimonas
	Fuso-e47b7c5	p. Fusobacteria; c. Fusobacteriia; o. Fusobacteriales; f. Fusobacteriaceae; g. Fusobacterium
	Fret-2b8622a	p. Synergistetes; c. Synergistia; o. Synergistales; f. Synergistaceae; g. Fretibacterium
	Solo-114d0ae	p. Firmicutes; c. Erysipelotrichia; o. Erysipelotrichales; f. Erysipelotrichaceae; g. Solobacterium
	Clos-a82bde6	p. Firmicutes; c. Clostridia; o. Clostridiales; o. Clostridiales; o. Clostridiales
	Camp-5b14d87	p. Proteobacteria; c. Epsilonproteobacteria; o. Campylobacterales; f. Campylobacteraceae; g. Campylobacter
	Bact-26547c9	p. Bacteroidetes; c. Bacteroidia; o. Bacteroidales; f. Bacteroidaceae; g. Bacteroides
	Gran-a5b69f1	p. Firmicutes; c. Bacilli; o. Lactobacillales; f. Carnobacteriaceae; g. Granulicatella
	Lach-7273e6e	p. Firmicutes; c. Clostridia; o. Clostridiales; f. Lachnospiraceae; g. Lachnospiraceae FCS020 group
	Porp-69023d0	p. Bacteroidetes; c. Bacteroidia; o. Bacteroidales; f. Porphyromonadaceae; g. Porphyromonas
	Fuso-7fa9543	p. Fusobacteria; c. Fusobacteriia; o. Fusobacteriales; f. Fusobacteriaceae; g. Fusobacterium
	Geme-7bd174e	p. Firmicutes; c. Bacilli; o. Bacillales; f. Family XI; g. Gemella
	Lach-3682830	p. Firmicutes; c. Clostridia; o. Clostridiales; f. Lachnospiraceae; g. Lachnospiraceae FCS020 group
	Bifi-37020b4	p. Actinobacteria; c. Actinobacteria; o. Bifidobacteriales; f. Bifidobacteriaceae; g. Bifidobacterium
	Fuso-d2f4ba7	p. Fusobacteria; c. Fusobacteriia; o. Fusobacteriales; f. Fusobacteriaceae; g. Fusobacterium
	Bact-605c17c	p. Bacteroidetes; c. Bacteroidia; o. Bacteroidales; f. Bacteroidaceae; g. Bacteroides
	Fret-fd4122f	p. Synergistetes; c. Synergistia; o. Synergistales; f. Synergistaceae; g. Fretibacterium
	Camp-7e4c8c9	p. Proteobacteria; c. Epsilonproteobacteria; o. Campylobacterales; f. Campylobacteraceae; g. Campylobacter
	Porp-1693b47	p. Bacteroidetes; c. Bacteroidia; o. Bacteroidales; f. Porphyromonadaceae; g. Porphyromonas
	Bifi-d1d2824	p. Actinobacteria; c. Actinobacteria; o. Bifidobacteriales; f. Bifidobacteriaceae; g. Bifidobacterium
	Rumi-aaa27b7	p. Firmicutes; c. Clostridia; o. Clostridiales; f. Ruminococcaceae; g. Ruminiclostridium 5
	Rumi-af67155	p. Firmicutes; c. Clostridia; o. Clostridiales; f. Ruminococcaceae; g. Ruminococcaceae UCG-005

Table S9. Tumor rPCA PC 2 associated features used in the ALR calculation

	ASV ID	Taxonomy
Long-term Survival	Osci-d425791	p. Firmicutes; c. Clostridia; o. Clostridiales; f. Ruminococcaceae; g. Oscillibacter
	Bifi-d1d2824	p. Actinobacteria; c. Actinobacteria; o. Bifidobacteriales; f. Bifidobacteriaceae; g. Bifidobacterium
	Bact-605c17c	p. Bacteroidetes; c. Bacteroidia; o. Bacteroidales; f. Bacteroidaceae; g. Bacteroides
	Lach-b61ab87	p. Firmicutes; c. Clostridia; o. Clostridiales; f. Lachnospiraceae; f. Lachnospiraceae
	Bifi-37020b4	p. Actinobacteria; c. Actinobacteria; o. Bifidobacteriales; f. Bifidobacteriaceae; g. Bifidobacterium
	Alis-0bf0743	p. Bacteroidetes; c. Bacteroidia; o. Bacteroidales; f. Rikenellaceae; g. Alistipes
	Bact-b24ef5f	p. Bacteroidetes; c. Bacteroidia; o. Bacteroidales; f. Bacteroidaceae; g. Bacteroides
	Rose-f6bea81	p. Firmicutes; c. Clostridia; o. Clostridiales; f. Lachnospiraceae; g. Roseburia
Short-term Survival	Bact-47f3d64	p. Bacteroidetes; c. Bacteroidia; o. Bacteroidales; f. Bacteroidaceae; g. Bacteroides
	Bact-26547c9	p. Bacteroidetes; c. Bacteroidia; o. Bacteroidales; f. Bacteroidaceae; g. Bacteroides
	Bact-4abaa48	p. Bacteroidetes; c. Bacteroidia; o. Bacteroidales; f. Bacteroidaceae; g. Bacteroides
	Dial-35fd415	p. Firmicutes; c. Negativicutes; o. Selenomonadales; f. Veillonellaceae; g. Dialister
	Esch-ffc36e2	p. Proteobacteria; c. Gammaproteobacteria; o. Enterobacteriales; f. Enterobacteriaceae; g. Escherichia/Shigella
	Dial-63719d6	p. Firmicutes; c. Negativicutes; o. Selenomonadales; f. Veillonellaceae; g. Dialister
	Camp-5b14d87	p. Proteobacteria; c. Epsilonproteobacteria; o. Campylobacterales; f. Campylobacteraceae; g. Campylobacter
	Bact-b6635d6	p. Bacteroidetes; c. Bacteroidia; o. Bacteroidales; f. Bacteroidaceae; g. Bacteroides
	Fuso-fb12967	p. Fusobacteria; c. Fusobacteriia; o. Fusobacteriales; f. Fusobacteriaceae; g. Fusobacterium
	Bact-6251bd9	p. Bacteroidetes; c. Bacteroidia; o. Bacteroidales; f. Bacteroidaceae; g. Bacteroides
	Pept-f148c98	p. Firmicutes; c. Clostridia; o. Clostridiales; f. Peptostreptococcaceae; g. Peptostreptococcus
	Faec-4516aa6	p. Firmicutes; c. Clostridia; o. Clostridiales; f. Ruminococcaceae; g. Faecalibacterium
	Bact-7440aa8	p. Bacteroidetes; c. Bacteroidia; o. Bacteroidales; f. Bacteroidaceae; g. Bacteroides
	Fuso-e47b7c5	p. Fusobacteria; c. Fusobacteriia; o. Fusobacteriales; f. Fusobacteriaceae; g. Fusobacterium
	Fuso-7fa9543	p. Fusobacteria; c. Fusobacteriia; o. Fusobacteriales; f. Fusobacteriaceae; g. Fusobacterium
	Fuso-d2f4ba7	p. Fusobacteria; c. Fusobacteriia; o. Fusobacteriales; f. Fusobacteriaceae; g. Fusobacterium
	Faec-bbae6ed	p. Firmicutes; c. Clostridia; o. Clostridiales; f. Ruminococcaceae; g. Faecalibacterium
	Lach-02e1e97	p. Firmicutes; c. Clostridia; o. Clostridiales; f. Lachnospiraceae; g. Lachnospiraceae UCG-010
	Porp-1693b47	p. Bacteroidetes; c. Bacteroidia; o. Bacteroidales; f. Porphyromonadaceae; g. Porphyromonas
	Stre-d3829c5	p. Firmicutes; c. Bacilli; o. Lactobacillales; f. Streptococcaceae; g. Streptococcus
	Porp-69023d0	p. Bacteroidetes; c. Bacteroidia; o. Bacteroidales; f. Porphyromonadaceae; g. Porphyromonas
	Bact-70d55ba	p. Bacteroidetes; c. Bacteroidia; o. Bacteroidales; f. Bacteroidaceae; g. Bacteroides
	Fuso-62b2f8b	p. Fusobacteria; c. Fusobacteriia; o. Fusobacteriales; f. Fusobacteriaceae; g. Fusobacterium
	Parv-8f3c8cb	p. Firmicutes; c. Clostridia; o. Clostridiales; f. Family XI; g. Parvimonas
	Faec-c728ad6	p. Firmicutes; c. Clostridia; o. Clostridiales; f. Ruminococcaceae; g. Faecalibacterium
	Fret-2b8622a	p. Synergistetes; c. Synergistia; o. Synergistales; f. Synergistaceae; g. Fretibacterium
	Dial-08dda4f	p. Firmicutes; c. Negativicutes; o. Selenomonadales; f. Veillonellaceae; g. Dialister
	Fret-fd4122f	p. Synergistetes; c. Synergistia; o. Synergistales; f. Synergistaceae; g. Fretibacterium

Supplementary Table 1 - The STORMS checklist. An editable version for adaptation and inclusion in publications is available from https://stormsmicrobiome.org

Number	Item	Recommendation	Item Source	Additional Guidance	Yes/No/NA	Comments or location in manuscript
**Abstract**						
1.0	Structured or Unstructured Abstract	Abstract should include information on background, methods, results, and conclusions in structured or unstructured format.	STORMS		Yes	pg 1, abstract
1.1	Study Design	State study design in abstract.	STORMS	See 3.0 for additional information on study design.	Yes	pg 1, abstract
1.2	Sequencing methods	State the strategy used for metagenomic classification.	STORMS	For example, targeted 16S by qPCR or sequencing, shotgun metagenomics, metatranscriptomics, etc.	Yes	pg 1, abstract
1.3	Specimens	Describe body site(s) studied.	STORMS		Yes	Pg 1, abstract
**Introduction**						
2.0	Background and Rationale	Summarize the underlying background, scientific evidence, or theory driving the current hypothesis as well as the study objectives.	STORMS		Yes	Ln 3-28 (pg 2-3)
2.1	Hypotheses	State the pre-specified hypothesis. If the study is exploratory, state any pre-specified study objectives.	STORMS		Yes	
**Methods**						

3.0	Study Design	Describe the study design.	STORMS	Observational (Case-Control, Cohort, Cross-sectional survey, etc.) or Experimental (Randomized controlled trial, Non-randomized controlled trial, etc.). For a brief description of common study designs see: DOI: 10.11613/BM.2014.022<br><br>If applicable, describe any blinding (e.g. single or double-blinding) used in the course of the study.	Yes	Ln 30-35 (pg 5)
3.1	Participants	State what the population of interest is, and the method by which participants are sampled from that population. Include relevant information on physiological state of the subjects or stage in the life history of disease under study when participants were sampled.	STORMS	Examples of the population of interest could be: adults with no chronic health conditions, adults with type II diabetes, newborns, etc. This is the total population to whom the study is hoped to be generalizable to. The sampling method describes how potential participants were selected from that population.<br><br>If the participants are from a substudy of a larger study, provide a brief description of that study and cite that study.<br><br>Clearly state how cases and controls are defined.		Lines 30-53 (pg 4-5)

				An example of relevant physiological state might be pre/post menopausal for a vaginal microbiome study; examples of stage in the life history of disease could be whether specimens were collected during active or dormant disease, or before or after treatment.		
3.2	Geographic location	State the geographic region(s) where participants were sampled from.	MIxS: geographic location (country and/or sea,region)	Geographic coordinates can be reported to prevent potential ambiguities if necessary.	Yes	Lines 40-42
3.3	Relevant Dates	State the start and end dates for recruitment, follow-up, and data collection.	STORMS	Recruitment is the period in which participants are recruited for the study. In longitudinal studies, follow-up is the date range in which participants are asked to complete a specific assessment. Finally, data collection is the total period in which data is being collected from participants including during initial recruitment through all follow-ups.		Lines 40-42

3.4	Eligibility criteria	List any criteria for inclusion and exclusion of recruited participants.	Modified STROBE	Among potential recruited participants, how were some chosen and others not? This could include criteria such as sex, diet, age, health status, or BMI.<br><br>If there is a primary and validation sample, describe inclusion/exclusion criteria for each.	Yes	Lines 40-46
3.5	Antibiotics Usage	List what is known about antibiotics usage before or during sample collection.	STORMS	If participants were excluded due to current or recent antibiotics usage, state this here.<br><br>Other factors (e.g. proton pump inhibitors, probiotics, etc.) that may influence the microbiome should also be described as well.	Yes	Lines 40-46
3.6	Analytic sample size	Explain how the final analytic sample size was calculated, including the number of cases and controls if relevant, and reasons for dropout at each stage of the study. This should include the number of individuals in whom microbiome sequencing was attempted and the number in whom microbiome sequencing was successful.	STORMS	Consider use of a flow diagram (see template at https://stormsmicrobiome.org/figures). Also state sample size in abstract.<br><br>If power analysis was used to calculate sample size, describe those calculations.	Yes	Lines 51-53
3.7	Longitudinal Studies	For longitudinal studies, state how many follow-ups were conducted, describe sample size at follow-up by group or condition, and discuss any loss to follow-up.	STORMS	If there is loss to follow-up, discuss the likelihood that drop-out is associated with exposures, treatments, or outcomes of interest.	N/A	Nested case-control design with people categorized by follow up time; data was extracted from

						Swedish death records which are very complete
3.8	Matching	For matched studies, give matching criteria.	Modified STROBE	"Matched" refers to matching between comparable study participants as cases and controls or exposed / unexposed.<br><br>Indicate whether participants were individual or frequency matched and in what ratio were they matched (e.g. 1 case to 1 control).	N/A	N/A<br>No matching was applied
3.9	Ethics	State the name of the institutional review board that approved the study and protocols, protocol number and date of approval, and procedures for obtaining informed consent from participants.	STORMS		Yes	Lines 55 – 56
4.0	Laboratory methods	State the laboratory/center where laboratory work was done.	STORMS	Provide a reference to complete lab protocols if previously published elsewhere such as on protocols.io. Note any modifications of lab protocols and the reason for protocol modifications.	Yes	Lines 73-79; reference 21
4.1	Specimen collection	State the body site(s) sampled from and how specimens were collected.	MIxS: sample collection device or method; host body site	Use terms from the Uber-anatomy Ontology (https://www.ebi.ac.uk/ols/ontologies/uberon) to describe body sites in a standardized format.	Yes	Lines 43-44; Table S1

4.2	Shipping	Describe how samples were stored and shipped to the laboratory.	STORMS	Include length of time from collection to receipt by the lab and if temperature control was used during shipping.	Yes	Lines 40-42
4.3	Storage	Describe how the laboratory stored samples, including time between collection and storage and any preservation buffers or refrigeration used.	STORMS	State where each procedure or lot of samples was done if not all in the same place.<br><br>Include reagent/lot/catalogue #s for storage buffers.	Yes	Ref 20
4.4	DNA extraction	Provide DNA extraction method, including kit and version if relevant.	MIxS: nucleic acid extraction	If any DNA quantification methods were used prior to DNA amplification or at the pooling step of library preparation, state so here.	Yes	Ref 20
4.5	Human DNA sequence depletion or microbial DNA enrichment	Describe whether human DNA sequence depletion or enrichment of microbial or viral DNA was performed.	STORMS		Yes	Ref 20
4.6	Primer selection	Provide primer selection and DNA amplification methods as well as variable region sequenced (if applicable).	MIxS: pcr primers		Yes	Line 108
4.7	Positive Controls	Describe any positive controls (mock communities) if used.	STORMS	If used, should be deposited under guidance provided in the 8.X items.	N/A	
4.8	Negative Controls	Describe any negative controls if used.	STORMS	If used, should be deposited under guidance provided in the 8.X items.	N/A	
4.9	Contaminant mitigation and identification	Provide any laboratory or computational methods used to control for or identify microbiome contamination from the environment, reagents, or laboratory.	STORMS	Includes filtering of reagents and other steps to minimize contamination. It is relevant to state whether the	N/A	

				specimens of interest have low microbial load, which makes contamination especially relevant.		
4.10	Replication	Describe any biological or technical replicates included in the sequencing, including which steps were replicated between them.	STORMS	Replication may be biological (redundant biological specimens) or technical (aliquots taken at different stages of analysis) and used in extraction, sequencing, preprocessing, and/or data analysis.	N/A	
4.11	Sequencing strategy	Major divisions of strategy, such as shotgun or amplicon sequencing.	MIxS: sequencing method	For amplicon sequencing (for example, 16S variable region), state the region selected. State the model of sequencer used.	Yes	Lines 73-79
4.12	Sequencing methods	State whether experimental quantification was used (QMP/cell count based, spike-in based) or whether relative abundance methods were applied.	STORMS	These include read length, sequencing depth per sample (average and minimum), whether reads are paired, and other parameters.	N/A	See ref 20
4.13	Batch effects	Detail any blocking or randomization used in study design to avoid confounding of batches with exposures or outcomes. Discuss any likely sources of batch effects, if known.	STORMS	Sources of batch effects include sample collection, storage, library preparation, and sequencing and are commonly unavoidable in all but the smallest of studies.	N/A	All samples were sequenced in the same batch
4.14	Metatranscriptomics	Detail whether any mRNA enrichment was performed and whether/how retrotranscription was performed prior to sequencing. Provide size range of isolated transcripts. Describe whether the sequencing library was stranded or not. Provide details on sequencing methods and platforms.	STORMS	Provide details on any internal standards which may have been used as well as parameters and versions of any software or databases used.	N/A	

4.15	Metaproteomics	Detail which protease was used for digestion. Provide details on proteomic methods and platforms (e.g. LC-MS/MS, instrument type, column type, mass range, resolution, scan speed, maximum injection time, isolation window, normalised collision energy, and resolution).	STORMS	Provide details on any internal standards which may have been used as well as parameters and versions of any software or databases used.	N/A	
4.16	Metabolomics	Specify the analytic method used (such as nuclear magnetic resonance spectroscopy or mass spectrometry). For mass spectrometry, detail which fractions were obtained (polar and/or non-polar) and how these were analyzed. Provide details on metabolomics methods and platforms (e.g. derivatization, instrument type, injection type, column type and instrument settings).	STORMS	Provide details on any internal standards which may have been used as well as parameters and versions of any software or databases used.	N/A	
5.0	Data sources/ measurement	For each non-microbiome variable, including the health condition, intervention, or other variable of interest, state how it was defined, how it was measured or collected, and any transformations applied to the variable prior to analysis.	MIxS: host disease status	State any sources of potential bias in measurements, for example multiple interviewers or measurement instruments, and whether these potential biases were assessed or accounted for in study design.<br><br>Use terms from a standardized ontology such as the Experimental Factor Ontology (https://www.ebi.ac.uk/efo/) to describe variables of interest in a standardized format.	Yes	Lines 45-46, Lines 60-70

6.0	Research design for causal inference	Discuss any potential for confounding by variables that may influence both the outcome and exposure of interest. State any variables controlled for and the rationale for controlling for them.	STORMS	For causal inference, this item refers to describing the assumptions that would be required to draw causal inferences from observational data. See Vujkovic-Cvijin, I., Sklar, J., Jiang, L. et al. Host variables confound gut microbiota studies of human disease. Nature 587, 448–454 (2020). https://doi.org/10.1038/s41586-020-2881-9 for more details on confounding in observational microbiome studies.<br><br>For example, hypothesized confounders may be controlled for by multivariable adjustment. Consider using a directed acyclic graph (DAG) to describe your causal model and justify any variables controlled for. DAGs can be made using www.dagitty.net.	Yes	Table S1, Table S2, Figure S3
6.1	Selection bias	Discuss potential for selection or survival bias.	STORMS	Selection bias can occur when some members of the target study population are more likely to be included in the study/final analytic sample than others. Some examples include survival bias (where part of the target study population is more likely to die before they can		Lines 40-46;

				be studied), convenience sampling (where members of the target study population are not selected at random), and loss to follow-up (when probability of dropping out is related to one of the things being studied).		
7.0	Bioinformatic and Statistical Methods	Describe any transformations to quantitative variables used in analyses (e.g. use of percentages instead of counts, normalization, rarefaction, categorization).	STORMS	If a variable is analyzed using different transformations, state rationale for the transformation and for each analyses which version of the variable is used.<br><br>In case of any complex or multistep transformations, give enumerated instructions for reproducing those transformations.	Yes	Lines 96-100; 103-105; 112-114; 118-125; 128-131.
7.1	Quality Control	Describe any methods to identify or filter low quality reads or samples.	MIxS: sequence quality check	If samples were excluded based on quality or read depth, list the criteria used, the number of samples excluded, and the final sample size after quality control.		Lines 48-53, Lines 90-92;
7.2	Sequence analysis	Describe any taxonomic, functional profiling, or other sequence analysis performed.	MIxS: feature prediction; similarity search method			Lines 81-92

7.3	Statistical methods	Describe all statistical methods.	Modified STROBE	Describe any statistical tests used, exploratory data analysis performed, dimension reduction methods/unsupervised analysis, alpha/beta metrics, and/or methods for adjusting for measurement bias.<br><br>If multiple statistical methods are possible, discuss why the methods used were selected.<br><br>If a multiple hypothesis testing correction method was used, describe the type of correction used.<br><br>State which taxonomic levels are analyzed.		Lines 60-70; 145-170
7.4	Longitudinal analysis	If the study is longitudinal, include a section that explicitly states what analysis methods were used (if any) to account for grouping of measurements by individual or patterns over time.	STORMS			Lines 102-108; 127-143;
7.5	Subgroup analysis	Describe any methods used to examine subgroups and interactions.	STROBE		N/A	
7.6	Missing data	Explain how missing data were addressed.	STROBE	"Missing data" refers to participant measurements such as covariates, exposures, outcomes, or time points that should have been collected but were not, not to zeros in taxonomic abundance tables or data	N/A	We had a case-complete dataset

				points not applicable to that observation.		
7.7	Sensitivity analyses	Describe any sensitivity analyses.	STROBE		N/A	
7.8	Findings	State criteria used to select findings for reporting.	STORMS	For example, false discovery rate with total number of tests, effect size threshold, significance threshold, microbes of interest.	Yes	Lines 118-125; 139-143; 165
7.9	Software	Cite all software (including read mapping software) and databases (including any used for taxonomic reference or annotating amplicons, if applicable) used. Include version numbers.	Modified STREGA	Installed packages, add-ons or libraries should be stated and cited in addition to the software used.<br><br>All parameters employed that differ from the default of that software/version should be provided.<br><br>This is in addition to, not a replacement for, publishing of code as outlined in the section Reproducible Research.	Yes	Lines 81; 85-92; 99-100; 103-104; 111-112; 136-137; 166-171

8.0	Reproducible research	Make a statement about whether and how others can reproduce the reported analysis.	STORMS	Any protected information that has been excluded or provided under controlled access should be listed along with any relevant data access procedures. "On request from authors" is not sufficiently detailed; formal data access procedures and conditions should be defined.<br><br>If data are unavailable, state so clearly.<br><br>Consider using a specialized rubric for reproducible research (such as: https://mbio.asm.org/content/9/3/e00525-18.short).<br><br>Consider preregistering the study protocol (such as on osf.io or https://plos.org/open-science/preregistration/).	Yes	See code and data avalaibility (lines 362-367)
8.1	Raw data access	State where raw data may be accessed including demultiplexing information.	STORMS	Robust, long-term databases such as those hosted by NCBI and EBI are preferred. If using a private repository, provide rationale.		ENA accession PRJEB57580

8.2	Processed data access	State where processed data may be accessed.	STORMS	Unfiltered data should be provided.<br><br>Robust, long-term databases such as those hosted by NCBI and EBI-EMBL are preferred. Repositories like zenodo (https://zenodo.org/) or publisso (https://www.publisso.de/en/working-for-you/doi-service/) can be used to provide a DOI and long-term storage for processed datasets, even those which cannot be published openly.	N/A	doi: 10.5281/zenodo.7325431
8.3	Participant data access	State where individual participant data such as demographics and other covariates may be accessed, and how they can be matched to the microbiome data.	STORMS	If re-categorized, transformed, or otherwise derived variables were used in the analysis, these variables or code for deriving them should be provided.<br><br>Examples of how participant data can be matched to microbiome data are: using the same set of anonymized identifiers, or using different anonymized identifiers but providing a map.<br><br>Provided data should be sufficient to independently replicate the current analysis.	N/A	doi: 10.5281/zenodo.7325431 and ENA accession PRJEB57580

8.4	Source code access	State where code may be accessed.	STORMS	If a standard or formalized workflow was employed, reference it here.	N/A	See code and data availability statement; lines 362-367
8.5	Full results	Provide full results of all analyses, in computer-readable format, in supplementary materials.	STORMS	For example, any fold-changes, p-values, or FDR values calculated, provided as a spreadsheet.<br><br>Use a machine-readable, plain-text format such as csv or tsv.	Yes	See supplemental material
**Results**						
9.0	Descriptive data	Give characteristics of study participants (e.g. dietary, demographic, clinical, social) and information on exposures and potential confounders.	STROBE	Typically reported in a table included in the paper or as a supplementary table. Indicate number of participants with missing data for each variable of interest.<br><br>This includes environmental and lifestyle factors that may affect the relationship between the microbiome and the condition of interest. Participant diet and medication use should be summarized, if known.<br><br>At minimum, age and sex of all participants should be summarized.		Table S1

10.0	Microbiome data	Report descriptive findings for microbiome analyses with all applicable outcomes and covariates.	STORMS	This includes measures of diversity as well as relative abundances. These descriptive findings should be reported both for the sample overall and for individual groups.		Table S2, Figures S1, S3
10.1	Taxonomy	Identify taxonomy using standardized taxon classifications that are sufficient to uniquely identify taxa.	STORMS	If not using full taxonomic hierarchy, make sure it is clear whether names stated are species, genera, family, etc.<br><br>Italicize genus/species pairs. Consult journal guidelines or standardized references on taxonomic nomenclature. For instance, https://wwwnc.cdc.gov/eid/page/scientific-nomenclature	Yes;	Throughout manuscript
10.2	Differential abundance	Report results of differential abundance analysis by the variable of interest and (if applicable) by time, clearly indicating the direction of change and total number of taxa tested.	STORMS	If there are more than two groups, include omnibus (multigroup) test results if applicable to the research question.<br><br>If applicable, reported effect sizes should include a measure of uncertainty such as the confidence interval.	N/A	See ALR statistics; we. did not perform per-feature differential abundance
10.3	Other data types	Report other data analyzed--e.g. metabolic function, functional potential, MAG assembly, and RNAseq.	STORMS		N/A	

10.4	Other statistical analysis	Report any statistical data analysis not covered above.	STORMS	This could include subgroup analysis, sensitivity analyses, and cluster analysis.<br><br>Visualizations should be easily interpretable and colorblind-friendly. The caption and/or main text should provide a detailed description of visualizations for visually-impaired readers.	Yes	Figures 1-3; Figures S4; Table S3-S7
**Discussion**						
11.0	Key results	Summarise key results with reference to study objectives	STROBE		Yes	Lines 269-280
12.0	Interpretation	Give a cautious overall interpretation of results considering objectives, limitations, multiplicity of analyses, results from similar studies, and other relevant evidence.	STROBE	Define or clarify any subjective terms such as "dominant," "dysbiosis," and similar words used in interpretation of results.<br><br>When interpreting the findings, consider how the interpretation of the findings may be summarized or quoted for the general public such as in press releases or news articles.<br><br>If causal language is used in the interpretation (such as "alters," "affects," "results in," "causes," or "impacts"), assumptions made for causal inference should be	Yes	

				explicitly stated as part of 6.0 and 13.0.<br><br>Distinguish between function potential (ie inferred from metagenomics) and observed activity (ie metatranscriptomic, metabolomic, proteomic) if discussing microbial function.		
13.0	Limitations	Discuss limitations of the study, taking into account sources of potential bias or imprecision.	STROBE	Also consider limitations resulting from the methods (especially novel methods), the study design, and the sample size.	Yes	335-350
13.1	Bias	Discuss any potential for bias to influence study findings.	STORMS	May include sampling method, representativeness of study participants, or potential confounding.		335-350
13.2	Generalizability	Discuss the generalisability (external validity) of the study results	STROBE	To what populations or other settings do you expect the conclusions to generalize?		335-350
14.0	Ongoing/future work	Describe potential future research or ongoing research based on the study's findings.	STORMS			353-359
**Other information**						

15.0	Funding	Give the source of funding and the role of the funders for the present study and, if applicable, for the original study on which the present article is based	STROBE			371-377
15.1	Acknowledgements	Include acknowledgements of those who contributed to the research but did not meet critera for authorship.	STORMS	For general guidelines on authorship, see http://www.icmje.org and https://www.elsevier.com/authors/journal-authors/policies-and-ethics/credit-author-statement		364-370
15.2	Conflicts of Interest	Include a conflicts of interest statement.	STORMS			385-387
16.0	Supplements	Indicate where supplements may be accessed and what materials they contain.	STORMS			pg 32
17.0	Supplementary data	Provide supplementary data files of results with for all taxa and all outcome variables analyzed. Indicate the taxonomic level of all taxa.	STORMS	Depending on the analysis performed, examples of the supplemental results included could be mean relative abundance, differential abundance, raw p-value, multiple hypothesis testing-adjusted p-values, and standard error.<br><br>All discussed taxa should include the taxonomic level (e.g. class, order, genus).		See supplemental material

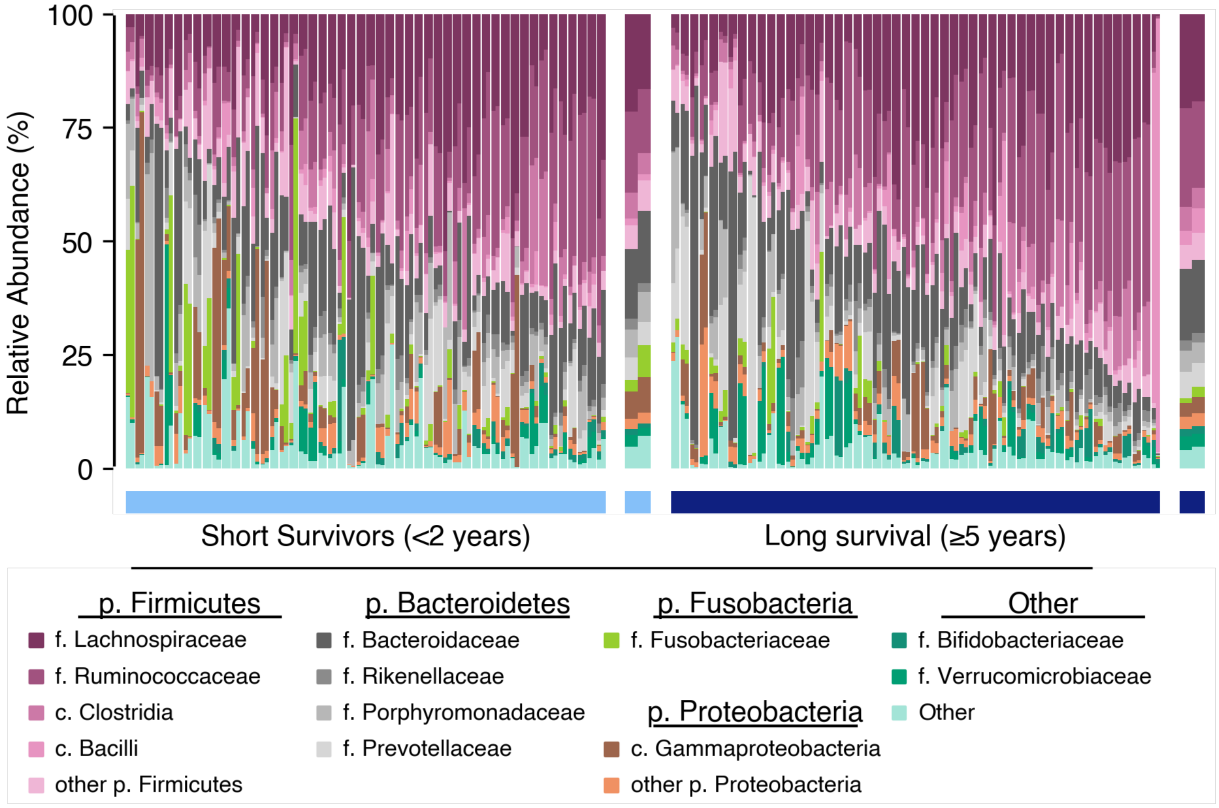

Figure S1. A family level view of the microbiome
Samples are sorted by individual donor with the normal tissue on the left and tumor on the right. Data is colored by taxonomic group (p: phylum level; c: class; f: family). The wide bars to the right of each distribution group show the average of the normal (left) and tumor (right) for all samples in the survival group.

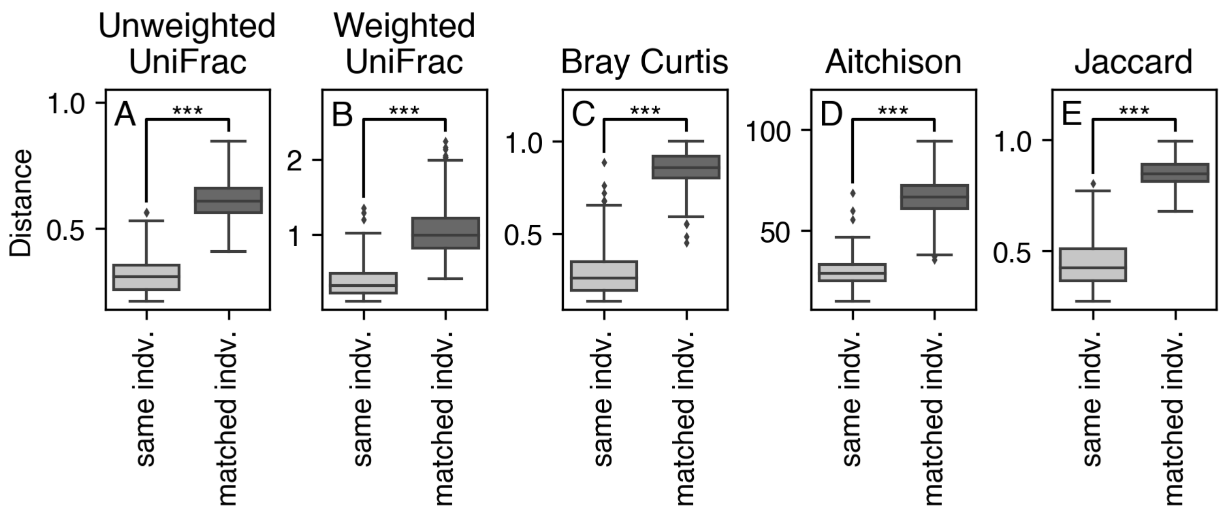

Figure S2. There is a strong individual effect on the microbiome
Beta diversity measured with (A) unweighted and (B) weighted UniFrac distances, (C) Bray Curtis dissimilarity, (D) Aitchison Distance, and (E) Binary Jaccard distance show within individual paired sample distances compared to the distance other samples matched on tissue type, anatomical location, surgery year, and survival. P-value from permutative t-test with 999 permutations; ***p=0.001, **p ≤ 0.01, *p ≤0.05.

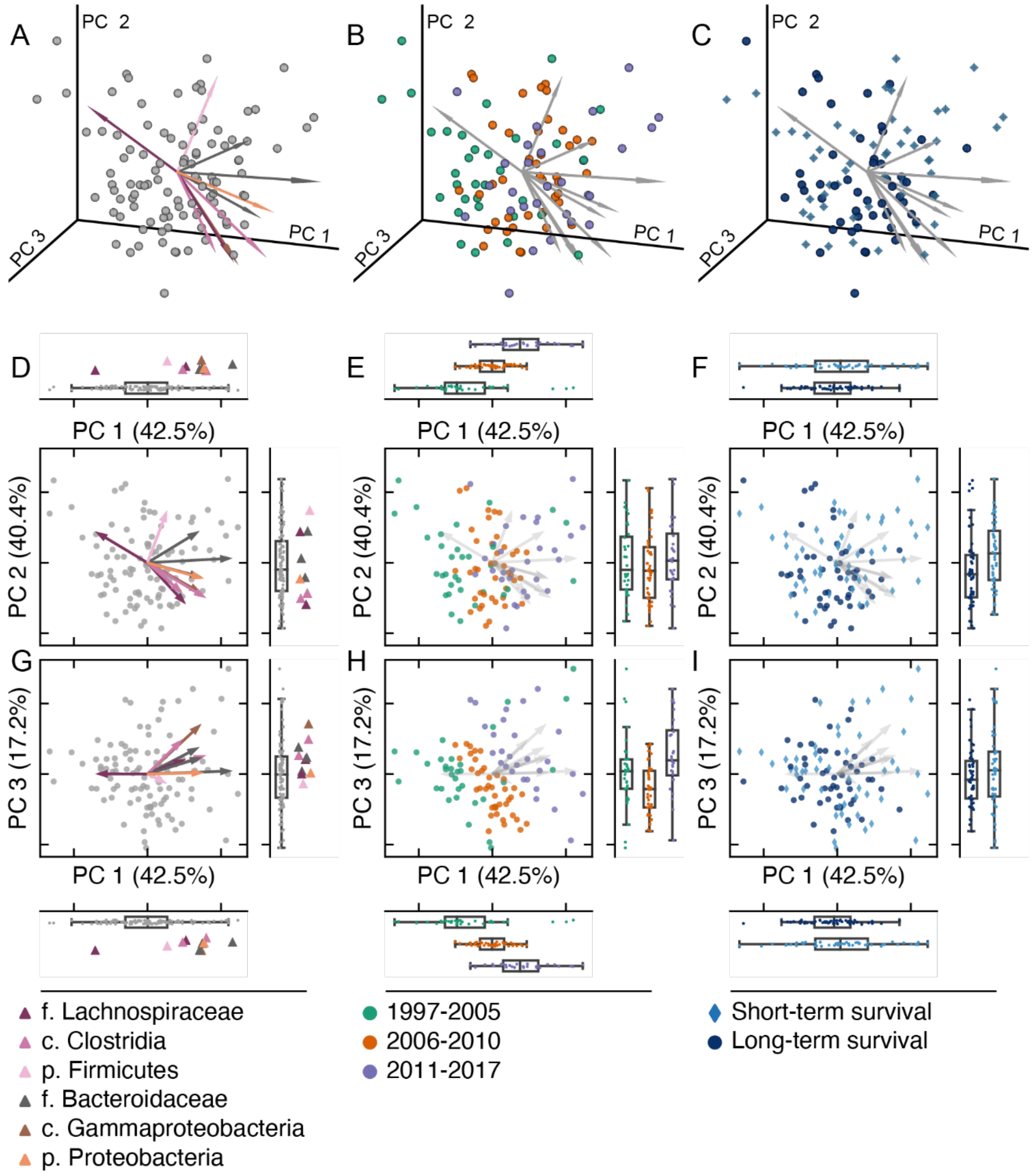

Figure S3. Metadata predictors of the microbiome in Compositional Tensor Factorization.

Ordination plots show (A-C) three-dimension PCA projection plots, (D-F) PC 1 vs PC 2 and (G-I) PC 2 vs PC 3 for the per-subject ordination. Plots show (A,D,G) biplot features, (B,E,H) the period of surgery, and (C,F,I) the survival. Marginal axes show the distribution of samples along their respective axes.

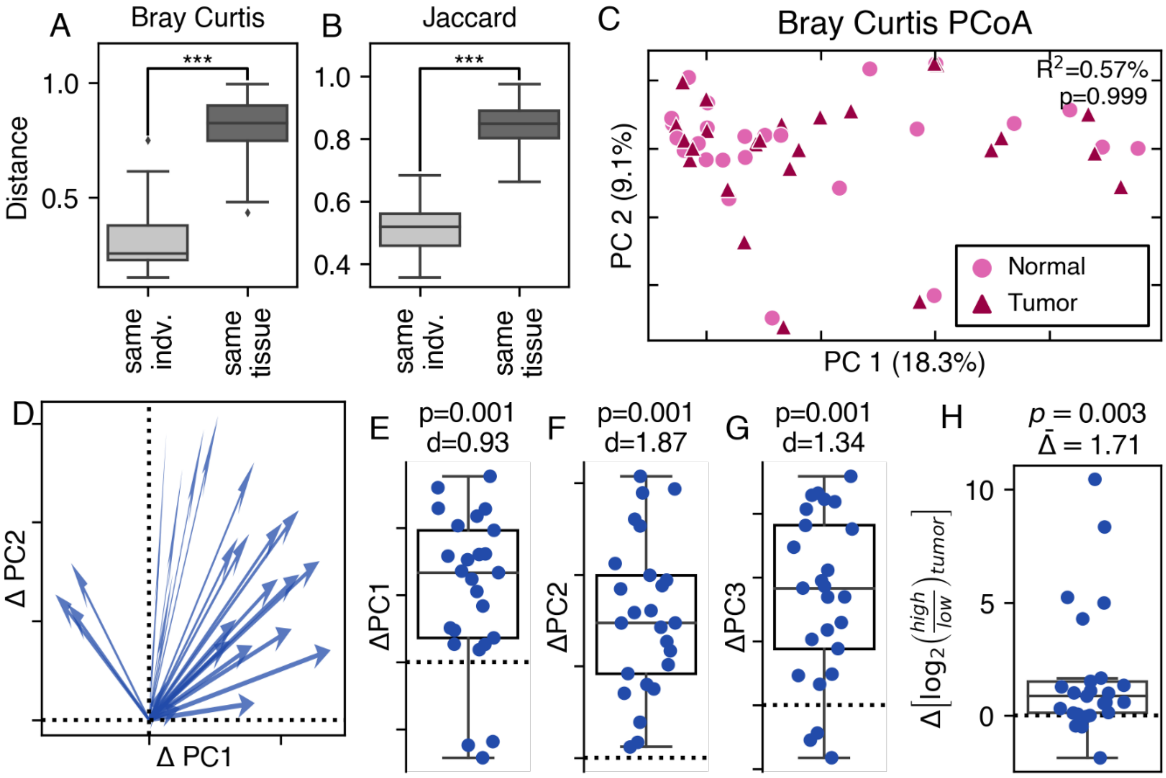

Figure S4. Subject aware techniques detect a consistent between tissue types and replicate associated taxa.
We compared paired samples from 25 Irish colorectal cancer patients. (A,B) Paired samples from the same individual had more similar microbiomes than samples of the same tissue (tumor or normal tissue) from other individuals. The difference was tested with a permutative t-test: *p≤.05, **p≤0.01, ***p=0.001, 999 permutations. (C) We performed a principal coordinates analysis to compare the global centroids of paired normal (light pink circles) and tumor (dark pink trianges) samples. The variance explained and p-value were calculated using an unadjusted permanova with 999 permutations. (D-G) The use of a subject-aware CTF ordination shows a clear difference in the microbiome between paired tissue samples from the same individual along all three PCs. (D) A common, directional change can be seen along PC 1 and PC2 when the difference between normal tissue and tumor tissue is plotted as a vector. The difference between normal- and tumor tissue can also be observed along individual components: (E) PC 1, (F) PC 2, and (G) PC 3. Ticks and dashed zero-lines along PC 1 (E) and PC 2 (F) match the two-dimensional axes in (D). All boxplots are shown with a Cohen's d effect size statistic for a one-sided t-test and p-values from a permutative one sample t-test, 999 permutations. (H) There is a change in the tissue associated ALR in our replication cohort between tissue types. The replication data was fit to reference sequences from the original cohort; the relationship between the tissue type and ALR was modeled using a linear mixed effects model treating the individual as a random intercept. The data is labeled with the estimated coefficient and parametric p-value.

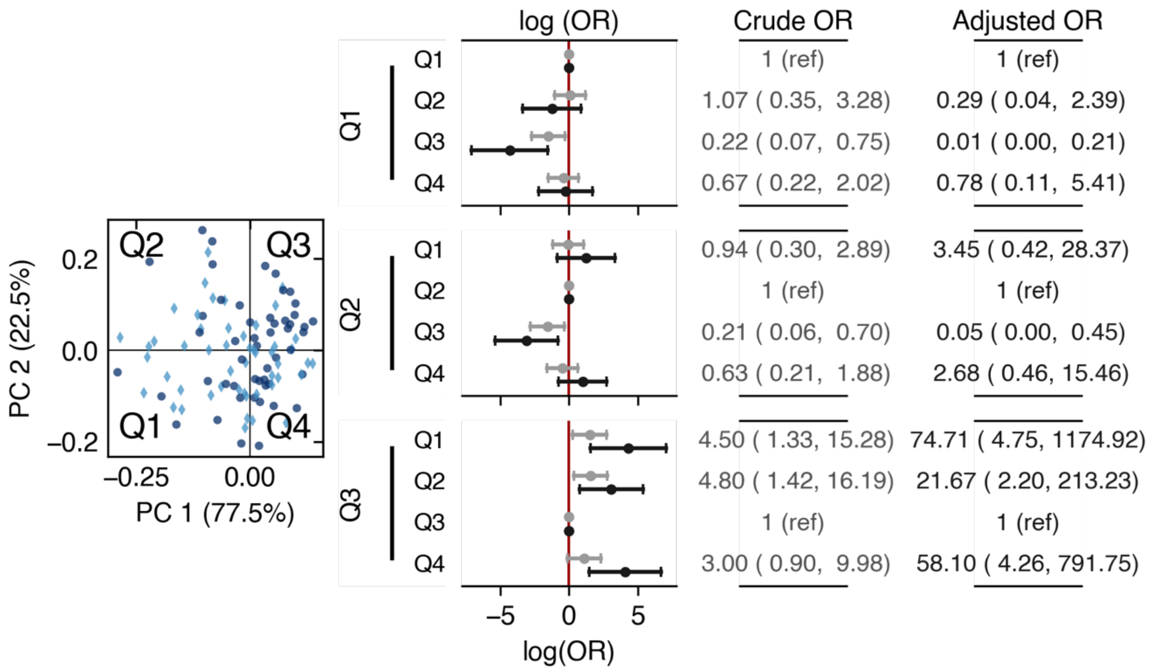

Figure S4. Survival separates individuals by tumor-associated rPCA quadrant
Left shows the quadrants in the rPCA space. Middle plot shows the log odds ratio (OR) for pairwise quadrants with 95% CI. Negative log OR indicates long survival, positive values indicate short survival. Red line indicates 0. Right shows the OR for the crude (light gray) and adjusted (dark gray) of short survival. The Adjusted OR is adjusted for age, sex, ASA score, tumor location, surgery year, TNM stage, and grade of differentiation.
